# A Retrospective Cohort Study on Hassab’s Surgery as a Salvage Treatment for Patients with Secondary Prophylaxis Failure for Acute Variceal Bleeding

**DOI:** 10.3390/jcm14248772

**Published:** 2025-12-11

**Authors:** Hongwei Zhang, Yuxue Xing, Danpu Wang, Rong He, Ke Zhang, Li Jiang, Zhe Jia

**Affiliations:** 1 Department of General Surgery, Di Tan Hospital, Beijing 100015, China; zhw084011@163.com (H.Z.); 18001152880@163.com (D.W.); 13810995338@163.com (R.H.); kezhang1130@163.com (K.Z.); jiangli1903@163.com (L.J.); 2 Department of Radiology, Di Tan Hospital, Beijing 100015, China; setsu220305@163.com

**Keywords:** acute variceal bleeding, secondary prophylaxis, portal hypertension, Hassab’s surgery, chronic liver disease

## Abstract

**Objective:** To investigate the safety and efficacy of Hassab’s surgery as a salvage treatment for patients with secondary prophylaxis failure for acute variceal bleeding (AVB), and to determine the role of Hassab’s surgery in the recompensation of cirrhosis and nutritional improvement. **Methods:** This study retrospectively analyzed data of 19 patients with AVB caused by cirrhosis and portal hypertension who underwent Hassab’s surgery as a salvage treatment after secondary prophylaxis failure in our center from March 2018 to June 2021. In addition, 47 patients with esophageal and gastric varices who underwent secondary prophylaxis during the same period were assigned to the control group to assess the safety and efficacy of the surgery. The objective laboratorial index and L3-SMA (the L3 skeletal muscle area, cm^2^, a radiological index for assessing whole-body skeletal muscle mass via CT measurement at the third lumbar vertebra level) of patients in the experimental group before and after surgery were compared to evaluate re-compensation of cirrhosis and nutritional improvement. **Results:** There was no significant difference in the incidence of perioperative complications and severe complications (Clavien–Dindo grade ≥ IIIb) between the experimental group and the control group. The 5-year re-bleeding-free survival rate and the 5-year overall survival rate in the experimental group were 73.7% and 94.7%, respectively, which were not significantly different from those in the control group. In addition, compared with before surgery, the white blood cell count, platelet count, hemoglobin level, model for end-stage liver disease (MELD) score, Child–Pugh grades, prothrombin time (PT), international normalized ratio (INR), and L3-SMA significantly increased in the experimental group after surgery. **Conclusions:** Hassab’s surgery proves to be a safe and effective salvage treatment for patients with AVB caused by liver cirrhosis and portal hypertension who failed to undergo secondary prophylaxis. Meanwhile, it was found that after surgery, not only were hypersplenism and coagulation abnormalities relieved, but also cirrhosis was compensated and nutritional status was improved significantly. Thus, this study revealed that Hassab’s surgery with safety and long-term survival effects can be used for patients with secondary prophylaxis failure for AVB in eligible patients

## 1. Introduction

Portal hypertension (PHT) refers to the increased resistance of blood vessels in the liver caused by impaired circulation of the hepatic sinuses, and it is known as the most common cause of chronic liver disease (CLD) [1,2]. Acute variceal bleeding (AVB) is one of the most common and life-threatening complications of PHT. Even one year after intervention, the rate of rebleeding is 60% and the mortality rate is 33% [3]. Therefore, secondary prophylaxis for AVB is vital. The relevant guidelines have recommended non-selective beta-blockers (NSBBs) and endoscopic therapies, including endoscopic variceal ligation (EVL) and endoscopic variceal obturation (EVO) [4].

The purpose of secondary prevention is to prevent recurrent venous bleeding in patients with a history of AVB. Failed secondary prevention of AVB is defined as recurrent variceal-related gastrointestinal bleeding in patients with a prior AVB history, despite adherence to standardized secondary prophylaxis (NSBBs and endoscopic therapies). A number of guidelines have recommended transjugular intrahepatic portosystemic shunt (TIPS) as salvage therapy [3,5,6]. In China, according to the relevant guidelines and expert consensus, both surgery and TIPS are recommended as salvage therapies for patients with AVB failing to undergo secondary prophylaxis [7,8]. Surgeries (e.g., Sugiura procedure, Hassab’s surgery, etc.), which are the main methods for secondary prophylaxis, have been gradually relegated to the secondary position in China due to the continuous emergence and improvement of various non-surgical approaches and the successful implementation of liver transplantation, although they have been reported to achieve accurate hemostasis and low incidence of serious complications [8]. At present, for patients with secondary prophylaxis failure, due to concerns about hepatic encephalopathy after TIPS, liver transplantation is not an ideal option, and Hassab’s surgery can be regarded as a salvage treatment. For such patients, it is essential to preoperatively indicate whether they meet the surgical requirements; if they could meet the surgical requirements, Hassab’s surgery has been shown to effectively prevent the recurrence of gastrointestinal bleeding, improving the liver function by preliminary clinical investigation [9]. The present study aimed to retrospectively analyze data of 19 patients with AVB caused by cirrhosis and PHT who underwent Hassab’s surgery as a salvage treatment in the Beijing Ditan Hospital, affiliated to Capital Medical University (Beijing, China), from March 2018 to June 2021, after secondary prophylaxis failure. The efficacy and safety of this treatment were evaluated to determine its clinical significance for patients with AVB who failed to undergo secondary prophylaxis.

## 2. Materials and Methods

### 2.1. Study Design

This study retrospectively analyzed data of 89 patients with AVB caused by cirrhosis and PHT who underwent Hassab’s surgery in Beijing Ditan Hospital, affiliated to Capital Medical University, from 1 March 2018 to 1 June 2021. Notably, 19 patients who were treated with Hassab’s surgery after secondary prophylaxis failure for AVB were assigned to experimental group, and 47 contemporaneous patients with a history of AVB who underwent Hassab’s procedure as secondary prevention were enrolled as the control group. The inclusion criteria is summarized as follows: (1) 18 < age ≤ 70 years; (2) PHT caused by liver cirrhosis and complicated by esophageal and gastric varices; (3) patients with hepatitis B virus infection who were treated with antiviral therapy preoperatively and regular medication to control viral load postoperatively, patients with hepatitis C virus infection who achieved sustained virological response after preoperative administration of direct-acting antiviral agents (DAAs), and patients with alcoholic cirrhosis at least 1 month before surgery; (4) no severe cardiac, pulmonary, renal, or metabolic diseases; and (5) no concurrent surgical procedures were performed. All enrolled patients were followed up by telephone until 1 September 2025 or death, whichever occurred earlier. This study was approved by the Ethics Committee of Beijing Ditan Hospital, affiliated to Capital Medical University and complied with the Declaration of Helsinki. (Approval NO.DTEC-KT2022-003-01). Written informed consent was obtained from all participants. Data were de-identified immediately after collection. All analyses were performed on de-identified data. Access to identifiable data was restricted to the principal investigator for essential study management purposes only.

### 2.2. Preoperative Management

Preoperative laboratory and related auxiliary examinations were completed. Electronic gastroscopy was performed to investigate varicose veins in the esophagus and fundus of the stomach, and surgical indications were defined based on the patient’s medical history and the above-mentioned inspection. Data of liver and portal venous system were collected by contrast-enhanced computed tomography (CE-CT) or contrast-enhanced magnetic resonance imaging (CE-MRI). Liver volume was measured using a three-dimensional (3D) reconstruction technique, and individualized Hassab’s surgery was designed. Child–Pugh score and model for end-stage liver disease (MELD) score were used to evaluate the liver function preoperatively. For patients with poor liver function, liver protection should be strengthened, hypoproteinemia should be corrected, and diuresis should be used to relieve ascites preoperatively. For patients with anemia, hemoglobin level should be corrected to >7 g/dL, and plasma transfusion and intramuscular vitamin K administration were performed to improve patients’ coagulation status. Cardiopulmonary function and risks of anesthesia and surgery were also assessed. All patients or their family members signed informed consent forms prior to surgery.

### 2.3. A 3D Reconstruction Technique Was Used to Design Individualized Hassab’s Surgery

Due to the repeated endoscopic interference, the paraesophageal veins might cause chronic or subacute inflammatory lesions in the surrounding tissue and local anatomical structure might change, leading to noticeable difficulties in intraoperative identification and separation of tissues and organs, as well as the increased risks of bleeding and collateral damage. A 3D reconstruction method was preoperatively used to clarify the distribution of spontaneous shunt and varicose veins after endoscopic therapy, and to develop an individualized surgical plan. Intraoperatively, the spleen was removed to reduce portal pressure, and the area with significant varicosity was dissected. The spontaneous portal shunt channels of the paraesophageal venous plexus and posterior peritoneum were preserved as far as possible.

### 2.4. Postoperative Management and Follow-Up

Postoperative vital signs, quality and volume of drainage fluid, and liver function were closely monitored, and abdominal Doppler ultrasound findings were periodically reviewed to investigate the hydrothorax or ascites and portal vein thrombosis. When the platelet count exceeded 100 × 10^9^/L and there was no bleeding tendency, anticoagulant therapy with low-molecular-weight heparin was carried out to prevent the occurrence or progression of portal vein thrombosis. Oral anticoagulant drugs were maintained after discharge, and the risk of bleeding after anticoagulant therapy was evaluated regularly, anticoagulation was discontinued when the platelet count returned to the normal range.

One month after surgery, patients were re-admitted to the hospital to postoperatively evaluate the recovery. Gastroscopy was performed again, and variceal ligation or tissue glue injection was conducted on the residual severe varices. Patients were followed up every 3–6 months according to their liver function, and patients without residual varicose veins were reexamined by gastroscopy once a year.

### 2.5. Measurement of L3 Skeletal Muscle Area (L3-SMA) Before and After Surgery

Using the medical image management system of Beijing Ditan Hospital, the original preoperative abdominal CT data of 19 patients in the experimental group were collected, as well as the postoperative CT data of 14 cases in the experimental group. The median postoperative CT follow-up time was 9.5 (range, 5–16) months. CT images of the third lumbar spine (L3) were imported into SliceOmatic image analysis software (version 6.0 Rev-9c2), and two specialists identified and quantified all skeletal muscles in images (including psoas major, erectus spinae, quadrates lumborum, transverus abdominis, internal and external oblique, and rectus abdominis) at Hounsfield unit (HU) range of −29 to +150. The total area of skeletal muscle in this layer was calculated by software to obtain L3-SMA. Finally, values achieved by the two specialists were averaged.

### 2.6. Statistical Analysis

Statistical analysis was performed using SPSS 20.0 software (IBM, Armonk, NY, USA). The collected data were classified. The measurement data were expressed as mean ± standard deviation or median (range), and categorical data were presented as counts and percentages. Before comparing two groups, the normality test was first performed and the homogeneity of variance of the two groups was assessed. Considering that the two groups of data did not conform to normal distribution or homogeneity of variance, the Mann–Whitney U test (a rank-sum test) was used for comparisons between groups. When the paired test was utilized for the analysis of data in the experimental group before and after surgery, the normality test (the Shapiro–Wilk (S–W) test) was utilized for comparing the differences in paired samples. The paired t-test was employed for the analysis of normally distributed data, and the Wilcoxon signed-rank test was used for the analysis of abnormally distributed data. Enumeration data (e.g., gender, etiology, preoperative related diseases, etc.) were analyzed by the Chi-square test or Fisher’s exact test, as appropriate.

It was assumed that the primary outcome of the study was recurrent gastrointestinal bleeding after surgery. The survival analysis was performed by the Kaplan–Meier method. Gastrointestinal bleeding was defined as the varicose hemorrhage of esophagus and gastric fundus caused by PHT with hematemesis or black stool and other clinical manifestations, which did not include postoperative bleeding of gumming ulcer caused by previous endoscopic treatment, as well as erosion bleeding of digestive mucosa caused by portal hypertensive gastropathy. Secondary outcomes were 1-year, 3-year, and 5-year mortality during follow-up. All data were obtained from electronic medical records and telephone follow-up. Patients were followed up after surgery until 1 September 2025 or death, whichever occurred earlier. *p* < 0.05 was considered statistically significant.

## 3. Results

### 3.1. Clinical Characteristics of Patients

Table 1 summarizes the clinical characteristics of patients in the two groups, and no significant difference was observed in any clinical characteristics between the two groups. Among 19 patients in the experimental group, there were 14 men and 5 women. Their median age was 46 (range, 27–64) years old. The causes of cirrhosis in these patients were hepatitis B virus infection in 13 (68.4%) patients, hepatitis C virus infection in 1 (5.3%) patient, and alcoholic liver disease in 5 (26.3%) patients. Laboratory examination after admission indicated that 12 (63.2%) cases had Child–Pugh grade A and 7 (36.8%) cases had Child–Pugh grade B for the first time after admission. Platelet count was 36 (13.0–74.0) × 10^9^/L, white blood cell count was 1.64 (1.10–10.04) × 10^9^/L. There were no significant differences in white blood cell count, hemoglobin level, platelet count, liver function, operation time, intraoperative blood loss, and other intraoperative conditions between the two groups.

### 3.2. Perioperative Complications

To enable standardized assessment, perioperative complications were stratified using the Clavien–Dindo classification, with detailed incidence rates (*n*, %) summarized in Table 2. Among the 19 patients in the experimental group, severe complications (defined as Clavien–Dindo grade ≥ IIIb) occurred in 5.3% (1/19) of cases; this single event corresponded to postoperative liver failure, diagnosed in accordance with the “50-50 criteria” [10]. Notably, no perioperative mortality was observed in the experimental group (0%, 0/19). For the control group (*n* = 47), the incidence of severe complications (Clavien–Dindo ≥ IIIb) was 4.3% (2/47), and perioperative mortality was recorded at 2.1% (1/47). Across all complication subtypes (stratified by Clavien–Dindo grade), no statistically significant differences in incidence were detected between the two groups (all *p* > 0.05; Table 2).

### 3.3. Comparison of Laboratory Indicators Before and After Surgery in the Experimental Group

As presented in Table 3, comparisons of the experimental group’s laboratory indicators (preoperatively vs. 3 months postoperatively) revealed prominent hematologic and coagulation improvements:

White blood cell count increased from a median (range) of 1.64 (1.1–10.04) × 10^9^/L to 5.98 (2.83–11.72) × 10^9^/L (*p* = 0.009).

Platelet count rose sharply from 36 (13.0–74.0) × 10^9^/L to 336.00 (67.20–792.10) × 10^9^/L (*p* < 0.001).

Hemoglobin levels improved from 85 (59–115) g/dL to 101 (71–129) g/dL (*p* = 0.033), with preoperative anemia largely resolved.

Coagulation function (INR) normalized from 1.42 (1.15–1.84) to 1.21 (0.99–1.84) (*p* = 0.006).

These changes were consistent with effective surgical management of hypersplenism.

**Figure 1:**
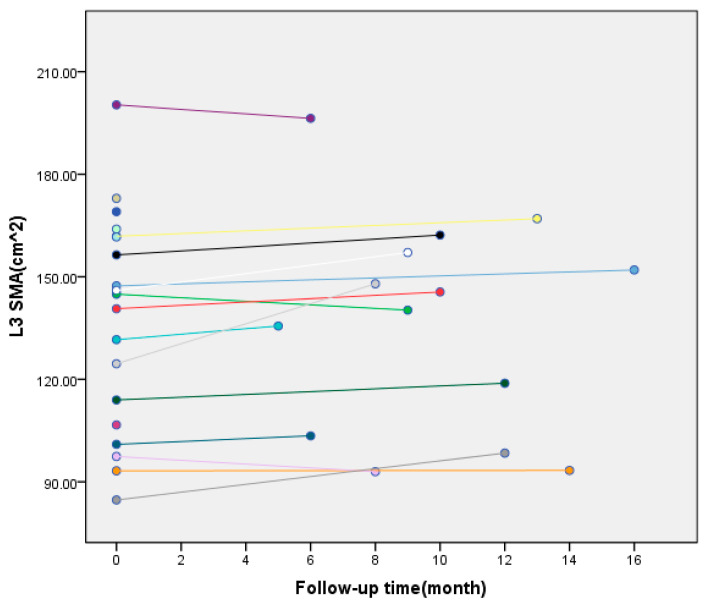
Changes in skeletal muscle index of third lumbar vertebrae (L3-SMA) of 19 patients in the experimental group before and after surgery (data of 5 patients were excluded because of loss of postoperative imaging data). Each colored dot corresponds to one of the 19 patients enrolled in the experimental group, whereas the lines illustrate the dynamic change trends of L3-SMA for each patient throughout the follow-up period.

Notable enhancements were further observed across other clinical domains:

Liver function: Child–Pugh score decreased from a median range of 6 (5–9) to 5 (5–7) (*p* = 0.033), while MELD score declined from 10 (2–15) to 8 (2–15) (*p* = 0.010);

Nutritional status: L3-SMA increased from 131.70 ± 31.75 cm^2^ to 136.50 ± 31.23 cm^2^ (median postoperative CT follow-up time: 9.5 months, range: 5–16 months) (*p* = 0.034) (Figure 1).

### 3.4. Re-Bleeding-Free Survival and Overall Survival (OS)

**Figure 2:**
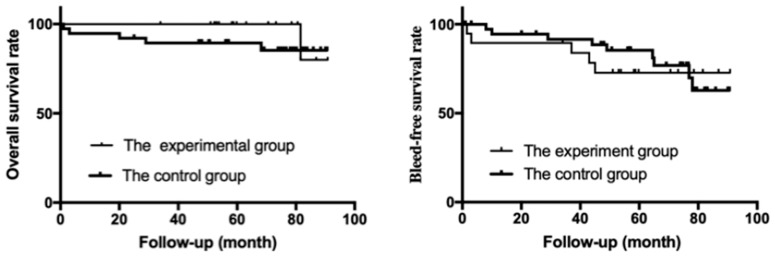
Kaplan–Meier curves of re-bleeding-free survival and overall survival in the two groups.

Long-term follow-up was performed in both groups postoperatively. The median follow-up time was 63 (range, 34–90) months in the experimental group and 74 (range, 1–90) months in the control group. The 5-year OS rate was 94.7% in the experimental group and 86.8% in the control group and with no significant difference between the two groups (*p* = 0.365) (Figure 2). During the follow-up period, one patient in the experimental group died of gastrointestinal bleeding. In the control group, five patients died during follow-up: two died of liver cancer, one died of rebleeding, one died of leukemia, and one died of cerebral hemorrhage. The 5-year re-bleeding-free survival rate in the experimental group and control group was 73.7% and 76.3%, respectively, and there was no significant difference between the two groups (*p* = 0.683) (Figure 2). Furthermore, five patients in the experimental group experienced gastrointestinal bleeding after surgery. Among them, two patients showed no rebleeding during the follow-up period after two weeks of endoscopic sclerotherapy; another two patients had improved conditions after conservative treatment and also had no rebleeding during follow-up. One patient still had a small amount of bleeding after conservative treatment, but no rebleeding occurred during the follow-up period after receiving two weeks of endoscopic sclerotherapy.

## 4. Discussion

If no preventive measures are taken for patients with PHT-induced AVB after treatment, the incidence of re-bleeding within 1–2 years is 60%, and the fatality rate is 33% [5]. To improve survival, secondary prophylaxis should be performed for patients recovering from AVB. Meanwhile, under the failure of combination therapy, including EVL and NSBBs, AASLD and KASL trials recommended TIPS as a salvage therapy [3,6]. However, the efficacy of surgery in this condition has not yet been clarified. According to the guidelines and expert consensus in China, both surgery and TIPS are recommended as rescue methods after secondary prophylaxis failure for AVB [7,8]. The surgical procedures include devascularization, shunt, or combination of devascularization and shunt. It has been proven that devascularization is effective (including Sugiura procedure, Hassab’s surgery, and modified Hassab’s surgery) in preventing bleeding and long-term protection of liver function [11]. Although the postoperative incidence of portal thrombosis is noticeable, several studies, including the present study, have confirmed that surgery is associated with an extremely low incidence of serious complications (e.g., intestinal necrosis, gastrointestinal bleeding, and liver failure) [12] However, in order to prevent the postoperative portal thrombosis, combination of devascularization and shunt is highly suggested as a more reasonable therapy. Due to the high requirements of shunt on splenic vessels and complex surgical techniques, devascularization has always been the main therapy for the prevention and treatment of gastrointestinal bleeding in PHT patients in China.

Owing to the individual differences in patients and the complexity of hemodynamics in PHT, some PHT patients still suffer from gastrointestinal bleeding after regular secondary prophylaxis; they cannot effectively control bleeding and typically suffer from bleeding, as well as physical, psychological, and economic burden and decreased quality of life. Moreover, due to the repeated gastrointestinal bleeding and aggravated damage of liver function and multiple organs, patients eventually suffer from failure of liver and multiple organs. Thus, after secondary prophylaxis failure, selection of an effective salvage treatment is important to prolong the survival and improve the quality of life. The present study showed that surgery, as a salvage therapy, could ensure patients’ safety under the premise of meeting the surgical conditions with satisfactory prognosis, and the postoperative re-bleeding rate could be remarkably reduced.

Baveno VII, renewing consensus in PHT, proposed the concept of “cirrhosis re-compensation” [13]. By reviewing cases in the experimental group, it was found that the majority of patients in the experimental group could meet the criteria required for “cirrhosis compensation” after surgery, in which the main causes of cirrhosis were eliminated, suppressed, or cured, including the elimination of hepatitis C virus, continuous inhibition of hepatitis B virus, and abstaining from alcohol in patients with alcoholic cirrhosis. During the postoperative follow-up (34–90 months) in the experimental group, except for five cases with recurrent bleeding (no re-bleeding occurred after endoscopy or drug treatment), other patients had no recurrent AVB. Only two patients had ascites when reexamined at 3 months after surgery. No hepatic encephalopathy occurred in all patients before and after surgery. At 3 months after surgery, there were only two patients with ascites. No hepatic encephalopathy occurred before and after surgery. Compared with before surgery, liver function, metabolism, and other functions were significantly improved. According to the above-mentioned results, it could be concluded that patients with cirrhosis after devascularization were compensated.

To our knowledge, about 40% of patients with compensatory cirrhosis were malnourished. When the disease developed to decompensation stage, 65–90% of patients were under protein-energy malnutrition (PEM), accompanied by changes in body composition, decreasing adipose tissue and somatic cells, and insufficient protein and/or caloric supply to maintain normal physiological function, leading to multiple organ dysfunction and poor prognosis [14,15]. Improvement in the nutritional status of cirrhotic patients, particularly skeletal muscle mass recovery, inherently requires a certain time course. Hassab’s surgery contributes to this process through two key mechanisms: (1) alleviating hypersplenism via splenectomy, which reduces the destruction of red blood cells, white blood cells, and platelets, thereby improving systemic anemia and immune dysfunction to lay the foundation for nutrient absorption and metabolism, and (2) mitigating the impact of portal hypertension on gastrointestinal circulation, which alleviates gastrointestinal congestion and edema while enhancing intestinal mucosal barrier function and the absorption efficiency of nutrients (e.g., proteins, amino acids). These pathophysiological changes may align temporally with our median follow-up period of 9.5 months. Notably, shorter follow-up durations (e.g., 3–6 months) may only capture initial trends of nutritional improvement, whereas longer periods (e.g., 9–12 months) are more conducive to observing significant skeletal muscle mass accumulation. Although our results at the median follow-up time indicate significant nutritional improvement, variability in follow-up duration may introduce bias. First, patients with short-term follow-up (<6 months) may have unresolved postoperative stress responses (e.g., surgical trauma and inflammatory factor release), leading to unstable nutrient absorption and utilization, which could result in lower L3-SMI elevation compared to those with long-term follow-up—potentially underestimating the surgery’s long-term nutritional benefits if this subgroup is large. Conversely, patients with long-term follow-up may exhibit selection bias (e.g., milder baseline conditions and better compliance), with their nutritional improvement potentially influenced by confounding factors such as inherent baseline status and postoperative management rather than the surgery alone. Second, cirrhotic patients’ nutritional status is significantly affected by liver function compensation and complications (e.g., ascites and hepatic encephalopathy); longer follow-up increases the risk of fluctuations in these factors, which may interfere with the direct association between L3-SMI changes and surgical efficacy. Hassab’s surgery is associated with nutritional improvement in cirrhotic patients at the median 9.5-month follow-up, but the potential impact of follow-up duration heterogeneity cannot be fully excluded. Future studies should adopt standardized follow-up protocols to further verify the reliability of these conclusions.

At present, TIPS is recognized as an important salvage therapy after failure of secondary prophylaxis. Holster et al. demonstrated that TIPS was superior to the combination of NSBB and EVL in reducing re-bleeding of varicose veins, while it could not prolong patients’ survival. In addition, the application of TIPS stents was found to be associated with a higher incidence of hepatic encephalopathy [16,17]. Thus, advantages and disadvantages of TIPS as a salvage therapy after failure of secondary prophylaxis remain to be further clarified.

For cirrhosis-induced decompensated events, the 5-year mortality rate of patients with variceal bleeding, as an independent complication, was 20%, while the 5-year mortality rate of patients with other complications, such as PHT, could reach more than 80% [18]. Only the management of esophageal variceal bleeding was previously emphasized; however, it is necessary to improve OS rate and quality of life. Consideration of only certain complications is not suggested. On the contrary, during management of varicose veins in the esophagus and fundus, varicose veins or other cirrhosis-related complications after variceal bleeding should be considered together [19]. The present study suggested the safety and effectiveness of devascularization for patients failing to undergo secondary prophylaxis for AVB. More importantly, the liver function, coagulation function, hyperplenism, and nutritional status of patients affected by cirrhosis were improved postoperatively, and recompensation of cirrhosis was even found in patients, thereby improving patients’ long-term survival rate and quality of life.

## 5. Conclusions

Notably, standardized diagnostic and therapeutic guidelines for cirrhotic esophageal variceal bleeding are still lacking, because there are no comprehensive studies that can aid in risk assessment and no prospective clinical trials to assist clinicians in making clinical decisions [20]. In conclusion, this study demonstrated that individualized treatment with safety and ideal long-term effects can be performed for some patients with recurrent gastrointestinal bleeding complicated with PHT who failed to undergo secondary prophylaxis on the premise of meeting surgical requirements. At present, surgeries other than transplantation are excluded from the prevention and treatment of AVB in Western countries, such as European countries and the United States. We believe that it is inappropriate to adopt this approach in regions with an imbalance between liver transplantation supply and demand.

This study was conducted retrospectively in a single institution with a small sample size, so the statistical results of this experiment have certain limitations. Relevant clinical cases should be collected and long-term follow-up should be conducted on a larger patient population to further validate research results and accumulate more clinical experience.

## Figures and Tables

**Table 1:** Demographic and clinical characteristics of patients.

Characteristic	Experimental Group (*n* = 19)	Control Group (*n* = 47)	Statistic	*p* Value
Age, years old, median (range)	46 (27–64)	49 (23–70)	−0.057	0.955
Sex, male, *n* (%)	14 (73.7)	36 (76.6)	0.000	1.000 *
Etiology			0.176	1.000
HBV infection, *n* (%)	13 (68.4)	32 (68.1)		
HCV infection, *n* (%)	1 (5.3)	3 (6.4)		
Alcoholic liver disease, *n* (%)	5 (26.3)	12 (25.0)		
History of smoking, Y, *n* (%)	8 (42.1)	16 (34.0)	0.380	0.538
History of drinking, Y, *n* (%)	5 (26.3)	10 (21.3)		0.748 *
Laboratory tests after admission				
White blood cell count, median (range) × 10^9^/L	1.64 (1.1–10.04)	2.18 (0.94–12.03)	−1.282	0.200
Platelet count, median (range) × 10^9^/L	36 (13.0–74.0)	48.5 (15.4–169.0)	−0.432	0.666
Hemoglobin, median (range) g/dL	85 (59–115)	89 (58–136)	−0.637	0.524
Prothrombin activity, median (range) %	59 (43–75)	63 (41–89)	−0.057	0.955
INR, median (range)	1.42 (1.15–1.84)	1.38 (1.10–1.94)	−0.716	0.474
Child–Pugh classification, A/B/C, *n* (%)	12 (63.2)/7 (36.8)/0 (0)	31 (66.0)/14 (29.8)/2 (4.3)	0.729	0.885 *
MELD score	10 (2–15)	10 (1–16)	−0.107	0.915
Operation time, median (range) minutes	180 (100–390)	162.5 (90–420)	−0.724	0.469
Blood loss, median (range) mL	200 (100–900)	200 (50–1500)	−0.169	0.866

* Fisher’s exact test.

**Table 2:** Clavien–Dindo classification of perioperative complications.

Clavien–Dindo Classification	Complication	Experimental Group *n* (%)	Control Group *n* (%)	Statistic	*p* Value
I					
	Body temperature ≥38.5 °C	8 (42.1)	28 (59.6)	1.665	0.276
	Infection of incisional wound	3 (15.8)	8 (17.0)	-	1.000 *
	Ascites (>500 mL/day)	8 (42.1)	24 (51.1)	0.510	0.592
II					
	Portal vein thrombosis	9 (47.4)	12 (25.5)	2.974	0.143
	Gastroparesis	1 (5.3)	0 (0)	-	0.288 *
	Abdominal infection	1 (5.3)	7 (14.9)	-	0.422 *
III					
IIIa	Abdominal bleeding (no surgery needed to stop bleeding)	1 (5.3)	0 (0)	-	0.288 *
IIIb	Abdominal bleeding (Conservative treatment failed and surgery was needed to stop bleeding)	0 (0)	1 (2.1)	-	1.000 *
	Disruption of incisional wound	0 (0)	1 (2.1)	-	1.000 *
IV					
Iva	Postoperative liver failure	1 (5.3)	2 (4.3)	-	1.000 *
IVb	Multiple organ dysfunction	0 (0)	1 (2.1)	-	1.000 *
V					
	Death	0 (0)	1 (2.1)	-	1.000 *

Note: Complications were graded according to the Clavien–Dindo classification. Some patients had multiple concurrent complications. * Fisher’s exact test.

**Table 3:** Comparison of laboratory indicators in the experimental group before and after surgery.

Laboratory Indicators	Before Surgery	3 Months After Surgery	Statistic	*p* Value
White blood cell count, median (range) × 10^9^/L	1.64 (1.1–10.04)	5.98 (2.83–11.72)	−2.627	0.009
Platelet count, median (range) × 10^9^/L	36 (13.0–74.0)	336.00 (67.20–792.10)	−3.621	<0.001
Hemoglobin, median (range) g/dL	85 (59–115)	101 (71–129)	−2.131	0.033
INR, median (range)	1.42 (1.15–1.84)	1.21 (0.99–1.84)	−2.757	0.006
Ascites, *n* (%)	5 (26.3)	2 (10.5)	-	0.405
Child–Pugh score, median (range)	6 (5–9)	5 (5–7)	−2.138	0.033
MELD score, median (range)	10 (2–15)	8 (2–15)	−2.586	0.010

## Data Availability

The datasets used and/or analyzed during the current study available from the corresponding author on reasonable request.
